# Breastfeeding and diseases prevalent in the first two years of a child’s life: a cross-sectional study

**DOI:** 10.1590/0034-7167-2021-0534

**Published:** 2022-06-06

**Authors:** Evelin Matilde Arcain Nass, Sonia Silva Marcon, Elen Ferraz Teston, Luciana Pedrosa Leal, Sueli Mutsumi Tsukuda Ichisato, Beatriz Rosana Gonçalves de Oliveira Toso, Mariana Angela Rossaneis Moreira, Fabiane Blanco Silva Bernardino

**Affiliations:** I Universidade Estadual de Maringá. Maringá, Paraná. Brazil.; II Universidade Federal de Mato Grosso do Sul. Campo Grande, Mato Grosso do Sul. Brazil.; III Universidade Federal de Pernambuco. Recife, Pernambuco. Brazil.; IV Universidade Estadual do Oeste do Paraná. Cascavel, Paraná. Brazil.; V Universidade Estadual de Londrina. Londrina, Paraná. Brazil.; VI Universidade Federal de Mato Grosso. Cuiabá, Mato Grosso. Brazil.

**Keywords:** Breast Feeding, Integrated Management of Childhood Illness, Comprehensive Health Care, Education, Nursing, Child Health Services, Lactancia Materna, Atención Integrada a las Enfermedades Prevalentes de la Infancia, Atención Integral de Salud, Enfermería Perioperatoria, Servicios de Salud Infantil, Aleitamento Materno, Atenção Integrada às Doenças Prevalentes na Infância, Assistência Integral à Saúde da Criança, Enfermagem Pediátrica, Serviços de Saúde Infantil

## Abstract

**Objectives:**

to assess the association between breastfeeding and diseases prevalent in the first two years of a child’s life.

**Methods:**

a retrospective cross-sectional study that analyzed electronic medical records of 401 children. Data on birth, growth, breastfeeding and medical care in the first two years of life were collected. In the analysis, Poisson regression with robust variance was used.

**Results:**

27.9% of children were exclusively breastfed until six months, and, at 24 months, 93.3% had already had some prevalent childhood disease. In the crude analysis, 5-minute Apgar association, length, weight at 12 months, exclusive and non-exclusive breastfeeding time had association. In the adjusted analysis, only the variable breastfeeding at six months maintained the association with prevalent childhood diseases.

**Conclusions:**

children who were not breastfed, exclusively or not, up to six months of age, had a higher prevalence of diseases compared to breastfed children.

## RESUMO

**Objetivos:**

avaliar a associação do aleitamento materno e as doenças prevalentes nos primeiros dois anos de vida da criança.

**Métodos:**

estudo transversal retrospectivo, que analisou prontuários eletrônicos de 401 crianças. Foram coletados dados sobre nascimento, crescimento, aleitamento materno e atendimentos médicos nos dois primeiros anos de vida. Na análise, utilizou-se Regressão de Poisson com variância robusta.

**Resultados:**

receberam aleitamento exclusivo até os seis meses 27,9% das crianças, e, aos 24 meses de vida, 93,3% já haviam tido alguma doença prevalente da infância. Na análise bruta, apresentaram associação Apgar no 5º minuto, comprimento, peso aos 12 meses, tempo de aleitamento exclusivo e não exclusivo. Na análise ajustada, apenas a variável aleitamento materno aos seis meses manteve a associação com as doenças prevalentes da infância.

**Conclusões:**

as crianças que não foram amamentadas, exclusivamente ou não, até os seis meses, apresentaram maior prevalência de doenças em relação às amamentadas.

## RESUMEN

**Objetivos:**

evaluar la asociación entre lactancia materna y enfermedades prevalentes en los dos primeros años de vida del niño.

**Métodos:**

estudio transversal retrospectivo que analizó las historias clínicas electrónicas de 401 niños. Se recogieron datos sobre nacimiento, crecimiento, lactancia y atención médica en los dos primeros años de vida. En el análisis se utilizó la regresión de Poisson con varianza robusta.

**Resultados:**

el 27,9% de los niños fueron amamantados exclusivamente hasta los seis meses de edad y, a los 24 meses, el 93,3% ya había tenido alguna enfermedad infantil prevalente. En el análisis crudo presentaron asociación de Apgar al minuto 5, longitud, peso a los 12 meses, tiempo de lactancia materna exclusiva y no exclusiva. En el análisis ajustado, sólo la variable lactancia materna a los seis meses mantuvo la asociación con las enfermedades prevalentes de la infancia.

**Conclusiones:**

los niños que no fueron amamantados, exclusivamente o no, hasta los seis meses de edad, presentaron mayor prevalencia de enfermedades en comparación con los niños amamantados.

## INTRODUCTION

The Integrated Management of Childhood Illness (IMCI) strategy, developed by the World Health Organization (WHO), the Pan American Health Organization and the United Nations Children’s Fund, aims to reduce morbidity and mortality in children between two months and five years of age^(1)^, by improving the quality of care offered by Primary Care^(2)^.

One of the main actions recommended by the IMCI regarding health promotion is performing exclusive breastfeeding (EBF) in the first six months of life and supplemented until the age of two years or more, since breast milk (BM) is an important protective factor for children’s health, being related to prevention of anemia, strengthening of the immune system, reduction of cases of infection, diarrhea and malnutrition^(3)^ and even infant mortality^(4)^.

With regard to prevalent diseases, a cohort study conducted in Sweden, with the objective of assessing the association between breastfeeding and hospitalizations for infectious diseases in children up to four years of age, revealed that the risk of hospitalizations for infectious diseases decreased with EBF duration. In early childhood, breastfeeding was associated with a decreased risk of enteral and respiratory infections and, in children aged two to four years, with a lower risk of respiratory infections^(5)^.

A study, whose objective was to assess the determinants of diarrhea in children aged 0 to 23 months in the city of Dessie, northeast of Ethiopia, showed that the reduction of acute diarrheal disease among children under two years old focuses on EBF improvement^(6)^.

Despite the notoriety regarding the benefits of EBF and its outcomes, strengthening the current evidence about the occurrence of prevalent childhood diseases in the first two years of life and their association with the protection provided by breastfeeding may reiterate the hypothesis that those babies who were breastfed will have greater protection compared to those who did not receive EBF. Thus, the results of this study may provide the possibility for nurses to discuss and support women who wish to breastfeed, since the theoretical and practical subsidies produced will provide examples of the direct relationship between breastfeeding and protection against some diseases, in addition to implementing safe interventions that will allow the improvement of measures to promote and protect breastfeeding.

Child health care in Brazil, as one of the priorities in the context of public policies, has undergone an extensive construction process throughout history, starting from a model centered on the disease and on curative actions to another based on a broader view of health, focusing on preventive actions and health promotion and protection, in which nurses’ practice is anchored^(5)^. Moreover, the second and fourth axes of the Brazilian National Policy for Comprehensive Child Health Care (PNAISC - *Política Nacional de Atenção Integral à Saúde da Criança*) guide breastfeeding (BF) and healthy complementary feeding, and comprehensive care for children with diseases prevalent in childhood and with chronic diseases, respectively^(6)^.

However, it is noteworthy that, even with the progress in relation to survival and child health in developing countries, such as Brazil, socioeconomic inequality is still present and has been accentuated in recent years, which is a determining factor in the health-infant disease process and influence on actions to prevent illness and deaths from preventable causes in childhood^(7)^. In this context, studies that repeatedly assess the influence of BF on the promotion of children’s health collaborate to direct public policies and their own professional practices, based on recent and reliable evidence.

## OBJECTIVES

To assess the association of BF and the diseases prevalent in the first two years of a child’s life.

## METHODS

### Ethical aspects

The study was carried out in accordance with the recommendations of Resolution 466/2012 of the Brazilian National Research Ethics Commission (CONEP *- Comissão Nacional de Ética em Pesquisa*). Its project was authorized by the Municipal Health Department and approved by the signatory institution’s Ethics Committee, which authorized the waiver of signing the Informed Consent Form, for using secondary data.

### Study design*, period, and location

This is a cross-sectional study, with matrix research, carried out in two hospitals that deliver by the Unified Health System (SUS - *Sistema Único de Saúde*) in the city of Maringá, PR, southern Brazil, with only one of them being Baby-Friendly Hospital. The matrix study aimed to assess gestational weight gain and postpartum weight retention and possible relationship with children’s living conditions and health. For the elaboration and description of this study, the Strengthening the Reporting of Observational studies in Epidemiology (STROBE)^(8)^ guidelines were used.

### Population and sample; inclusion and exclusion criteria

The study population consisted of children of mothers who participated in the matrix research. In the sample size delimitation, we considered the prevalence of 30.2% of weaning before 180 days of life^(9)^, the number of 5157 children born in 2017 in the living birth information system (SINASC-DATASUS)^(10)^, the 5% significance level, the 95% confidence interval and the 5% error, which resulted in a sample of 334 children who, plus 20% for possible losses and discontinuity, totaling 401 children.

The study included medical records of children living in Maringá, PR, who were born with gestational age ≥ 37 weeks and who, in the immediate puerperium, were in EBF. Twins, infants who discontinued EBF before hospital discharge, and children who, at the time of data collection, had not yet completed 24 months of life were not included.

### Study protocol

Data were collected from March to October 2020, by consulting the electronic medical records of children in the Management System of the Municipal Health Department. This integrated system is used by all the city’s Basic Health Units, which allowed the participants to be located from the information obtained in the matrix research.

Consultation and data collection in the management system was carried out by a single person (main researcher) who, through prior scheduling, appeared for eight months, twice a week, in the CECAPS room - Health Workers Training and Training Assistance - of the Health Department and accessed the system with the authorization of the sector.

The data of interest were those related to food, growth and medical care provided to children in the city’s health services.

The following exposure variables were considered:

- sociodemographic characteristics: sex (female, male); race/color (white, yellow, brown, black).
- birth data: 1- and 5-minute Apgar (score); birth weight and length (using growth curves from zero to two years according to sex (boys/girls), and considered adequate weight for age the standard z-score ≥-2 and ≤ +2 and inadequate weight when high for age (> + 2), low for age (≥-3 and <-2) and very low for age (<-3); appropriate length for age, z-score pattern ≥-2 and ≤ +2 and inappropriate length when high for age (> + 2), low for age (≥-3 and <-2) and very low for age (<-3), as recommended by the WHO^(11)^.
- growth data: body weight at 12 and 24 months (parameters adopted in the growth curves^(3)^.
- living and health conditions - food: EBF up to six months (only BM, directly from the breast or expressed, or human milk from another source, with no other liquids or solids, with the exception of drops or syrups containing vitamins, oral rehydration salts, mineral supplements or medications)^(3)^; BF at 12 and 24 months (BM direct from breast or milked, regardless of receiving other foods)^(3)^; updated vaccination calendar at 24 months of age; hospitalization history (yes/no); attendance at early childhood education center (yes/no).

The primary outcome variable under investigation was the presence (record in the medical record) of prevalent childhood diseases in the first two years of life and their association with EBF, identified from the diagnosis recorded by the doctor in the medical record. This record is accompanied by the number identified in the International Classification of Diseases (ICD-10). Diagnoses with frequency ≥ 10.0% were considered for analysis, namely: ICD J06 - acute infection of areas; ICD 05 - cough; ICD H66 - otitis; ICD R50 - fever; ICD A09 - diarrhea and gastroenteritis; ICD R10 - abdominal and pelvic pain; ICD K59 - functional bowel disorder; ICD K21 - gastroesophageal reflux; ICD N39 - urinary tract disorder; and ICD L22 - diaper dermatitis. For categorization, “presence” was considered when the children presented at least one episode of the aforementioned diseases. The secondary outcomes were the association of food and growth and these diseases, both linked to WHO recommendations^(3)^.

### Analysis of results, and statistics

Statistical analyses were performed using the Statistical Package for the Social Sciences (SPSS), 21.0. First, we performed the bivariate analysis between the independent variables with the outcome variable prevalent diseases in the first two years of life. The Kolmogorov-Smirnov test was used to test data normality. Variables with p < 0.20 in the bivariate analysis were selected to compose the model adjusted by Poisson regression, using the stepwise method, with robust variance. Possible confounding variables were tested in the statistical model in order to explain the association of interest. The measure of association used was the prevalence ratio (PR) for both bivariate analysis and Poisson regression. For both analyses, a 5% significance level was adopted in Wald’s chi-square test, and p-value and 95% confidence interval (95% CI) were presented.

## RESULTS

Of the 401 children whose medical records were part of the study, most were male (57.6%) and were born with adequate weight (83.5%), but this percentage decreased over time, so that, at 12 months, 66.3% had adequate weight, and at 24 months, this percentage dropped to 44.6%. At two years of age, 92.8% of children had an updated vaccination schedule and 84.8% attended an early childhood education center.

Regarding feeding, 27.9% received EBF until six months, 83.8% maintained BF until 12 months and 44.1% until 24 months. Regarding health, 81.5% had already presented some health problem at 12 months and at 24 this percentage increased to 93.3%. Finally, at 24 months, 99 (24.7%) children had already experienced at least one episode of hospitalization.

Table 1 shows that there was a higher frequency of illness in children without EBF at six months and in those without BF and with inadequate weight at 24 months.

**Table 1:** Conditions of birth, growth and health according to the presence of diseases in the first two years of life of children born in Maringá, Parná, Brazil, 2020

Variables	Total (401)	Diseases in children under 6 months (96)	Diseases from 7 to 11 months (231)	Diseases from 12 to 24 months (374)	*p* value
	n %	n %	n %	n %	
Sex					
Female	170 42.4	41 24.1	93 54.7	159 93.5	0.6811
Male	231 57.6	55 23.8	138 59.7	215 93.1	
Race/color					
White/yellow	202 50.4	51 25.5	116 57.4	189 93.6	0.8875
Brown/black	199 49.6	45 22.6	115 57.8	185 93.0	
5-minute Apgar					
9 - 10	398 99.2	95 23.9	229 57.5	371 93.2	0.8687
7 - 8	3 0.8	1 33.3	2 66.7	3 100.0	
Birth weight					
Adequate	335 83.5	83 24.8	188 56.1	313 93.4	0.7728
Inadequate	66 16.5	13 19.7	43 65.1	61 92.4	
Birth length					
Adequate	319 79.6	75 23.5	174 54.5	292 91.5	0.5440
Inadequate	82 20.4	21 25.6	57 69.5	82 100.0	
EBF at 6 months					
Yes	112 27.9	18 16.1	52 46.4	86 76.8	0.6141
No	289 72.1	78 30.0	179 61.9	288 99.6	
BF at 12 months					
Yes	336 83.8	81 24.1	187 55.6	309 92.0	0.8184
No	65 16.2	15 23.1	44 67.7	65 100.0	
BF at 24 months					
Yes	177 44.1	37 20.9	95 53.7	151 85.3	0.9984
No	224 55.9	59 26.3	136 60.7	223 99.6	
Weight at 12 months					
Adequate	266 66.3	62 23.3	147 55.3	240 90.2	0.9892
Inadequate	135 33.7	34 25.2	84 62.2	134 99.3	
Weight at 24 months					
Adequate	179 44.6	38 21.2	96 53.6	154 86.0	0.9576
Inadequate	222 55.4	58 26.1	135 60.8	220 99.1	
Vaccine updated					
Yes	372 92.8	92 24.7	212 57.0	349 93.8	0.8551
No	29 7.2	4 13.8	19 65.5	25 86.2	
Attend early childhood education center					
Yes	340 84.8	84 24.7	191 56.2	317 93.2	0.8095
No	61 15.2	12 19.7	40 65.6	57 93.4	
Were hospitalized					
Yes	99 24.7	21 21.2	62 62.6	96 97.0	0.9309
No	302 75.3	75 24.8	169 55.9	278 92.0	

EBF - exclusive breastfeeding; BF - breastfeeding.

Table 2 shows the number of medical visits in the first two years of life, for childcare or health problems, according to EBF. It is observed that the absence or only one episode of prevalent disease is statistically associated with a higher frequency of EBF (p-value 0.0098), while the presence of six to 15 episodes is significantly associated with the absence of EBF until the sixth month of life (p-value= 0.0350).

**Table 2:** Medical care in the first two years of life, according to the type of breastfeeding at six months, Maringá, Paraná, Brazil, 2020

Medical care	Exclusive breastfeeding			
	Yes n %	No n %	Total n %	*p* value
Childcare	15 55.5	12 44.5	27 6.7	0.0009
Childcare + 01 episodes	23 42.6	31 57.4	54 13.5	0.0098
02 to 05 episodes of diseases	45 31.7	97 68.3	142 35.4	0.2140
06 to 10 episodes of diseases	15 14.7	87 85.3	102 25.4	0.0006
11 to 15 episodes of diseases	10 16.7	50 83.3	60 15.0	0.0350
≥ 16 episodes of diseases	04 25.0	12 75.0	16 4.0	0.7898
Total	112	289	401	

Table 3 shows the prevalent childhood diseases in the first two years of life, according to the type of BF in the first six months of life, in which it is observed that the proportion of children with EBF affected by some prevalent childhood disease was significantly lower, with the exception of cases of suppurative otitis media and other urinary tract disorders, whose p-value was not statistically significant in relation to EBF.

**Table 3:** Prevalent childhood diseases in the first two years of life, according to the type of breastfeeding at six months, Maringá, Paraná, Brazil, 2020

Prevalent diseases of childhood	Exclusive breastfeeding			*p* value
	Yes (112) n %	No (289) n %	Total (401) n %	
Gastrointestinal tract				
Presumed infectious diarrhea and gastroenteritis	20 17.9	152 52.6	172 42.9	0.0001
Gastroesophageal reflux disease	20 17.9	108 37.4	128 31.9	0.0002
Functional bowel disorders	8 7.1	81 28.0	89 22.2	0.0001
Respiratory tract				
Acute upper airway infections of multiple sites	23 20.5	112 38.7	135 33.7	0.0005
Cough	18 16.1	116 40.1	134 33.4	0.0001
Otitis media suppurative and unspecified	21 18.7	42 14.5	63 15.7	0.2978
Others				
Abdominal and pelvic pain	22 19.6	129 44.6	161 40.1	0.0001
Diaper dermatitis	16 14.3	123 42.6	139 34.7	0.0001
Fever of unknown origin	25 22.3	112 38.7	137 34.2	0.0019
Other urinary tract disorders	14 12.5	32 11.1	46 11.5	0.6874

It is noteworthy that in the medical records of 27 (6.7%) children, medical records were related only to childcare. In other words, these children did not seek public health services in the city due to complaints related to health complications.

Tables 4 and 5 show the Poisson Regression. It is observed that, in the crude analysis (Table 4), seven variables showed an association with the prevalent diseases, two of them related to birth characteristics (5-minute Apgar and altered birth length), the type of feeding (EBF at six months and BF at 12 and 24 months), weight at 12 months of life and vaccination status.

**Table 4:** Crude prevalence ratio of the presence of diseases in the 24 months of life, according to sociodemographic variables, birth, nutritional status, breastfeeding and immunization of children born in Maringá, Paraná, Brazil, 2020

Variables	Had prevalent disease in the 24 months		Crude PR	95% CI	*p* value
	Yes	%			
Sex					
Female	159	93.5	1	-	0.85
Male	215	93.1	0.99	0.94-1.04	
Race/color					
White/yellow	189	93.6	1	-	0.99
Brown/black	185	93.0	0.99	0.94-1.05	
1-minute Apgar					
Normal	364	93.3	1	-	0.78
Altered	10	90.9	0.97	0.82-1.15	
5-minute Apgar					
Normal	313	93.3	1	-	0.000*
Altered	30	100.0	1.07	1.04-1.09	
Birth weight					
Normal	313	93.4			0.77
Altered	61	92.4	0.99	0.24-1.06	
Birth length					
Normal	292	91.5			0.000*
Altered	82	100.0	1.08	1.05-1.12	
EBF up to 6 months					
Yes	112	76.7	1	-	0.000*
No	65	77.5	1.22	1.15-1.30	
BF up to 12 months					
Yes	309	92.0			0.000*
No	65	100.0	1.08	1.05-1.11	
BF up to 24 months					
Yes	152	85.5	1	-	
No	223	99.6	1.15	1.09-1.21	0.000*
Weight at 12 months					
Normal	240	90.2	1	-	0.000*
Altered	134	99.3	1.09	1.05-1.13	
Vaccination status					
Complete	349	93.8	1	-	0.000*
Incomplete	25	86.2	0.92	0.81-1.05	

* p significant value; CI - confidence interval; BF - breastfeeding; EBF - exclusive breastfeeding.

In the adjusted PR analysis (Table 5), it is observed that, regardless of any other variable analyzed, children who were not breastfed, exclusively up to six months or did not receive BM up to In the adjusted PR analysis (Table 5), it is observed that, regardless of any other variable analyzed, children who were not breastfed, exclusively up to six months or did not receive BM up to 12 months, had a higher prevalence of prevalent diseases in relation to those who were breastfed (PR>1; p-value <0.05).

**Table 5:** Adjusted prevalence ratio of the presence of diseases at six, 12 and 24 months according to variables type of breastfeeding at six, 12 and 24 months, 5-minute Apgar, weight at 12 months and altered birth length of children born in Maringá, Paraná, Brazil, 2020

Variables	Adjusted PR	95% CI	*p* value
Not exclusively breastfed up to 6 months	1.21	1.14-1.29	0.000*
Not breastfed until 12 months	1.10	0.996-1.01	0.020*
Not breastfed up to 24 months	0.99	0.990-1.01	0.544
5-minute Apgar altered		0.988-1.01	0.854
Inadequate weight at 12 months	1.01	0.99-1.047	0.182
Birth length altered	1.01	1.00-1.031	0.055

* p significant value; CI - confidence interval; PR - prevalence ratio.

## DISCUSSION

The results of the multiple analysis model reiterate the association between the duration of EBF less than six months and its maintenance until 12 months and the presence of diseases prevalent in childhood in the first two years of life. This fact corroborates the indication of maintaining EBF in the first six months, as recommended and reinforces the evidence that anchors the nurses’ guidelines in primary care for children’s health promotion.

Primary care nurses, based on these data, can demonstrate that the highest prevalence of diseases is associated with the absence of EBF at six months and its maintenance until 12 months of life, and thus, during nursing consultation and collective actions, in possession of the understanding of preventable complications, encourage BF practice and maintenance.

The low prevalence of EBF at six months in the study (27.9%) indicates the need, despite all investments and research in BF, to seek strategies to support families and nursing mothers that can increase comply with and maintain this practice.

To increase the prevalence of EBF, support to the family and the nursing mother should be present during the gestational period, in the puerperium and in the first years of children’ life. In this sense, it is necessary to consider that the children under study were born in one of the two hospitals in the city that perform deliveries (normal and cesarean sections) financed by SUS and that only one of them is certified as a Baby-Friendly Hospital. The hospital responsible for more than 70% of deliveries by SUS in the city did not have this certification at the time of data collection from the matrix study.

It is noteworthy that the benefits of BF involve the factors of decreased health spending, a 36% reduction in the risk of sudden death and 13% in world infant mortality, resulting in increased life expectancy and quality of life^(12)^. Given this, in nursing practice, discussing with women the challenges of BF, the myths and the possibilities of management through the difficulties experienced can favor greater adherence and permanence of women in this process.

It is noteworthy that the causes of diseases prevalent in childhood can be identified early, which reinforces the importance of childcare for the proper monitoring of children’s growth and development. Through it, health professionals, through systematic physical/clinical examinations, guidance to the family on specific care for each age and early identification of signs of the main diseases in childhood^(13)^, can recommend to the family the most effective actions, such as emphasizing the importance of maintaining EBF. It is noteworthy that, due to the ability to build bonds with patients, nurses have a unique opportunity to educate, support and motivate.

In the crude analysis of this study, 5-minute Apgar between seven and eight, inadequate birth length, absence of EBF up to six months, BF at six, 12 and 24 months and inadequate weight at 12 months were significantly associated with prevalent childhood diseases, but, in the adjusted analysis, the higher prevalence of diseases was significantly associated with absence of EBF at six months and its maintenance until 12 months of life.

The research showed that the prevalence of EBF up to six months identified was lower than that recommended by WHO^(3)^. This result supports the data of a study conducted in southern Brazil with children under two years of age, which found a prevalence of EBF of 20.6%^(14)^. However, BF rates at 12 and 24 months were above the values identified in previous national surveys (1986, 1996, 2006 and 2013), in which the prevalence of BF in the first year of life increased from 22.7% in 1986 to 45.4% in 2013, and at two years of age, around 25% between 1986 and 2006, reaching 31.8% in 2013^(15)^.

Food introduction has unparalleled importance in children’s growth and in the emergence of diseases, depending on the time when it is started and the types of food introduced. Early inadequate food supply may lead to the manifestation of diseases in childhood and also in adulthood^(16)^.

EBF maintenance up to six months and prolonged EBF should not be understood as a single responsibility of the mother. The performance of women’s social network members and the guidance of health professionals on the importance of BF since prenatal care are fundamental^(17)^. In care practice, in maternity hospitals and Basic Health Units, the simple incorporation of the conduct of guiding and performing breast milking and offering milked milk can contribute significantly to BF promotion and maintenance^(18)^. Likewise, during the process of nursing education, it is necessary to use different strategies that, in fact, bring future professionals closer to the practical reality in carrying out actions to encourage and maintain BF that go beyond theoretical knowledge. Thus, activities directed, for example, by simulation, can develop the ability to deal with more complex BF situations.

Adequate weight was present in more than half of children who received EBF, similar to a study carried out in Santa Catarina with 303 children, assessed two years after delivery, which identified that children who were not exclusively breastfed had a higher risk of developing excess body weight^(19)^.

With regard to health problems, the most frequent were those related to the digestive and respiratory systems, being characterized with a significantly lower frequency in children who received EBF. This result corroborates the cohort conducted with 6,861 children belonging to six clinical research centers in the United States and Europe, which identified a significantly reduced gastrointestinal and respiratory infectious episode among children in EBF^(20)^.

Regarding the digestive system disease in children who did not receive EBF, diarrhea and gastroenteritis may be related to the absence of adequate hygiene of bottles and other kitchen utensils, in addition to the possibility of food intolerance related to the type of milk offered^(21)^, which could be considered confounding variables, however they were not assessed in this study. Abdominal and pelvic pain, in turn, is influenced by the myths and taboos that follow BF, since mothers are culturally persuaded by the family and known to introduce liquids, such as water and teas, believing that children are thirsty and that their performance will reduce pain, and thus will calm them and make them sleep more^(22)^.

Functional bowel disorders are a characteristic disease of early dietary introduction, and this occurs due to the provision of solid foods, associated with a low fluid intake. It is noteworthy that the development of infant feeding may be a reflection of parents’ and caregivers’ eating habits^(23-24)^.

In relation to respiratory system diseases, acute upper respiratory tract infections, in most cases, are associated with the practices of mothers during BF and baby positioning. A study carried out in the Midwest Region of Paraná, with 60 mothers of children aged between four and 180 days (mean of 40 days), identified that, among the 49 babies with EBF, nine (18.4%) had upper airway infections or otitis, a much lower proportion than that found among the 11 without EBF, since seven of them (63.6%) manifested these episodes^(25)^.

Otitis media in children may be related to anatomical positioning, since the auditory tube in infants is in a more horizontal position. Thus, the physiology of sucking during BF differs from that which occurs during the supply of dairy drinks through the bottle, in which muscle contraction is reduced, with consequent sagging of the soft palate muscles, and thus milk enters through the oropharynx and reaches the Eustachian tube. Since artificial milk has no antibodies, such as BM, rapid proliferation of bacteria is favored^(25)^.

Hospitalization frequency in this study (24.7%) is similar to the rate found in the cohort study conducted in Rio Grande do Sul, with 4,231 children followed up with one, two, four and six years of life. During the first year of life, hospitalization frequency was 19.1%, observing that “influenza and pneumonia” and “chronic lower airway diseases” groups were present among the three main causes of hospitalization, and the “intestinal infectious diseases” group was ranked between the third and fifth positions^(26)^.

In Brazil, more than 3,000 annual deaths of children could be avoided if they were exclusively breastfed until six months of age. In financial terms, almost one and a half billion dollars could be saved for inadequate BF and preventable deaths, more than 42 million dollars, in treatment of diarrhea and acute respiratory infection/pneumonia in children, and more than 12 billion dollars could be optimized for cognitive losses due to lack of BF. In other words, beyond life, which is priceless, the economy resulting from the combination of health spending, mortality and cognitive losses would total more than 14 billion dollars, which reinforces the importance of BF for the society’s economy and quality of life^(27)^

### Study limitations

The use of secondary data is noteworthy as a limitation, since they are limited to the reliability of the information obtained, because typing in the system is performed in a decentralized manner and by the professional responsible for the service. However, its results reiterate the importance of emphasizing BF as a protective factor for the most frequent diseases in childhood, subsidizing reflections on the need for health professionals to make use of practical strategies to encourage it.

Another limitation concerns the fact that the data come from medical records, which is justified by the fact that disease diagnosis is a medical attribution. It is noteworthy that childcare consultations performed by nurses were not assessed in the study, because, in these, the focus is on nursing diagnoses and interventions/guidelines. However, it is important to consider that the medical record at the health unit includes data on multidisciplinary care, and a nurse, at least in the analyzed records, did not record activities performed regarding care actions and/or guidance. Thus, complications are often initially identified or reported to the nursing team, but this is not recorded in the medical records, which contributes to little visibility of nurses’ work in primary care.

### Contributions to nursing and health

The results indicate that in nurses’ practice, monitoring children’s growth and development in the context of primary care, through childcare consultations, remains essential to support and promote BF for infant nutrition, due to its relationship with health outcomes in the first years of life. This knowledge is essential to develop and direct interventions that aim to reduce morbidity and mortality and improve children’s health and quality of life.

The low prevalence of EBF at six months and the prevalence of infectious diseases in the first years of life, especially of the respiratory and gastrointestinal tracts, observed in this study, are elements that arouse the need to discuss quality of care for children and families in primary care. Diseases preventable through personal hygiene, food, the environment, reinforcement of appropriate feeding and immunization guidelines, among others, should be less and less frequent in the population. The training of nurses and the application of the neonatal and child IMCI strategy in primary care could contribute to this reduction.

## CONCLUSIONS

It was concluded that, regardless of any other variable analyzed, children who were not exclusively breastfed until six months and did not maintain BF until 12 months had a higher prevalence of diseases in relation to breastfed women.

## SUPPLEMENTARY MATERIAL

The research database was deposited in SCIELO Data: Arcain, Evelin, 2021, “*Amamentação e as doenças prevalentes nos primeiros dois anos de vida da criança: estudo transversal*”, https://doi.org/10.48331/scielodata.XPWWYD SciELO Data, DRAFT VERSION, UNF:6:rWHWyqc8p/Ef7BQh3BLfwQ== [fileUNF].

0034-7167-reben-75-06-e20210534-sup01

0034-7167-reben-75-06-e20210534-sup02

0034-7167-reben-75-06-e20210534-sup03

0034-7167-reben-75-06-e20210534-sup04
