# Supplementary material for: Breastfeeding and diseases prevalent in the first two years of a child’s life: a cross-sectional study
Source: Rev Bras Enferm. 2022 Jun 6;75(6):e20210534. doi: 10.1590/0034-7167-2021-0534 (PMC9728897; doi:10.1590/0034-7167-2021-0534)
Supplement: 0034-7167-reben-75-06-e20210534-sup04 [file 0034-7167-reben-75-06-e20210534-sup04.pdf]

Título – Banco de dados da pesquisa “2021-0534 Amamentação e as doenças prevalentes nos primeiros dois anos de vida da criança: estudo transversal”.

Informações para contato: Evelin Matilde Arcain Nass, Secretaria Municipal de Saúde de Sarandi. E-mail: evelinmarcain@gmail.com

- Data da Coleta de dados: março a outubro de 2020.

- Localização geográfica da coleta de dados: O banco contém dados sobre alimentação e condições de saúde de crianças menores de dois anos, nascidas em Maringá- Paraná – Brasil, no período de março a outubro de 2018.

- Palavras-chave usadas para descrever o tópico de dados: Aleitamento Materno; Atenção Integrada às Doenças Prevalentes na Infância; Assistência Integral à Saúde da Criança; Enfermagem Pediátrica; Serviços de Saúde Infantil.

- Informações de idioma: Português.

- Fontes de financiamento – o estudo não foi financiado e a pesquisadora teve bolsa de doutorado pela CAPES nos anos de 2018 e 2019.

<u>Visão geral de dados e arquivos</u>

Para cada nome de arquivo, uma breve descrição de quais dados ele contém:

Banco planilha 1 e 4 contém os dados: sociodemográficas, nascimento, estado nutricional, aleitamento materno, imunização e a presença de doenças nos dois primeiros anos de vida.

Banco planilha 2 contém os dados: número de atendimentos médicos nos dois primeiros anos de vida e o tipo de aleitamento aos seis meses.

Banco planilha 3 contém os dados: as doenças prevalentes da infância nos dois primeiros anos de vida e o tipo de aleitamento aos seis meses.

- Data em que o arquivo foi criado: 03 de novembro de 2021.

- Data(s) em que o(s) arquivo(s) foi(m) atualizado(s) (versão) e a natureza da(s) atualização(ões), se aplicável: 17 de novembro de 2021.

<u>Informações metodológicas</u>

- Descrição dos métodos para coleta ou geração de dados (incluir links ou referências a publicações ou outra documentação contendo projeto experimental ou protocolos usados): Os dados foram coletados mediante consulta ao prontuário eletrônico das crianças, localizadas a partir do nome da mãe no sistema gestor da Secretaria Municipal de Saúde, este sistema é utilizado de forma integrada por todas as unidades básicas de saúde do município.

- Descrição dos métodos usados para processamento de dados (descreva como os dados foram gerados a partir dos dados brutos ou coletados): Os dados foram digitados e

inseridos em planilha do programa Microsoft Excel 2010. As análises estatísticas foram realizadas no programa Statistical Package for the Social Sciences (SPSS) 21.0.

- Qualquer informação específica de software ou instrumento necessária para entender ou interpretar os dados, incluindo números de versão de software e hardware: Realizou-se a análise bivariada entre as variáveis independentes com a variável de desfecho doenças prevalentes nos dois primeiros anos de vida.

A normalidade dos dados foi testada por meio do teste de Shapiro Wilk e de Kolmogorov-Smirnov. Variáveis com valor de $p < 0,20$ na análise bivariada foram selecionadas para compor o modelo ajustado por regressão de Poisson, pelo método stepwise, com variância robusta.

Possíveis variáveis confundidoras foram testadas no modelo estatístico para explicar a associação de interesse. A medida de associação empregada foi à razão de prevalência (RP) tanto para a análise bivariada como para a regressão de Poisson. Para ambas as análises se adotou o nível de significância de 5% no teste qui-quadrado de Wald e foram apresentados o valor de p e o intervalo de confiança de 95% (IC95%).

- Definições de códigos ou símbolos usados para observar ou caracterizar baixa qualidade/questionável/outliers que as pessoas devem estar cientes: em todas as planilhas do Banco a condição "presença" foi identificada com o número 1 e a "ausência" com o número 2.

- Pessoas envolvidas na coleta, processamento, análise e/ou envio de amostras: a coleta de dados no sistema gestor foi realizada por uma única pessoa (primeira autora), o processamento foi realizado por uma autora com formação em estatística e na análise ocorreu a participação de todas as autoras.

<u>Informações específicas de dados</u>

Banco tabela 1 e 4

- Número de variáveis: 52.

- Número de casos ou linhas: 402.

- Lista de variáveis, incluindo nomes completos e definições (soletrar palavras abreviadas) de cabeçalhos de coluna para dados tabulares:
- Aleitamento materno exclusivo (AME) aos 6 meses;
- Presença de doença aos 6 meses;
- Presença de doença aos 24 meses;
- Aleitamento materno (AM) aos 12 meses;
- Aleitamento materno (AM) aos 24 meses;
- Sexo;
- Raça/cor;
- 5° minuto;
- Peso ao nascer;
- Peso aos 12 meses;
- Peso aos 24 meses;
- Comprimento ao nascer;
- Estado vacinal;

- Centro de Educação Infantil;
- Internamento hospitalar.
- Unidades de medida
- Aleitamento materno exclusivo (AME) aos 6 meses: 1 presença, 2 ausência;
- Presença de doença aos 6 meses: 1 presença, 2 ausência;
- Presença de doença aos 12 meses: 1 presença, 2 ausência;
- Presença de doença aos 24 meses: 1 presença, 2 ausência;
- Aleitamento materno (AM) aos 12 meses: 1 presença, 2 ausência;
- Aleitamento materno (AM) aos 24 meses: 1 presença, 2 ausência;
- Sexo: 1 feminino, 2 masculino;
- Raça/cor: 1 branco e amarelo, 2 pardo e preto;
- 5° minuto: 1 de 10 a 9 pontos, 2 de 8 a 7 pontos;
- Peso ao nascer: 1 adequado para idade, 2 inadequado;
- Peso aos 12 meses: 1 adequado para idade, 2 inadequado;
- Peso aos 24 meses: 1 adequado para idade, 2 inadequado;
- Comprimento ao nascer: 1 adequado para idade, 2 inadequado;

* peso e comprimento ao nascer (utilizadas as curvas de crescimento de zero a dois anos, segundo o sexo (meninos/meninas) e considerado peso adequado para a idade o padrão de escore-z $\geq$ -2 e $\leq$ +2 e peso inadequado quando elevado para a idade (> + 2), baixo para a idade ($\geq$ -3 e < -2) e muito baixo para idade (< -3); comprimento adequado para idade o padrão de escore-z $\geq$ -2 e $\leq$ +2 e comprimento inadequado quando elevado para a idade (> + 2), baixo para a idade ($\geq$ -3 e < -2) e muito baixo para idade (< -3).

- Estado vacinal: 1 completo, 2 incompleto;
- Centro de Educação Infantil: 1 frequenta, 2 não frequenta;
- Internamento hospitalar: 1 presença, 2 ausência.

- Definições de códigos ou símbolos usados para registrar dados ausentes: número 2 refere-se a ausência

Banco tabela 2

- Número de variáveis: 7.

- Número de casos ou linhas: 402.

- Lista de variáveis, incluindo nomes completos e definições (soletrar palavras abreviadas) de cabeçalhos de coluna para dados tabulares:
- Aleitamento materno exclusivo (AME) aos 6 meses;
- Puericultura;
- 01 episódio de atendimento médico;
- 02 a 05 episódios de atendimento médico;
- 06 a 10 episódios de atendimento médico;
- 11 a 15 episódios de atendimento médico;
- 16 ou mais episódios de atendimento médico.

- Unidades de medida
- Aleitamento materno exclusivo (AME) aos 6 meses: 1 presença, 2 ausência;
- Puericultura: 1 realizada, 2 não realizada;
- 01 episódio de atendimento médico: 1 presença, 2 ausência;
- 02 a 05 episódios de atendimento médico: 1 presença, 2 ausência;

- 06 a 10 episódios de atendimento médico: 1 presença, 2 ausência;
- 11 a 15 episódios de atendimento médico: 1 presença, 2 ausência;
- 16 ou mais episódios de atendimento médico: 1 presença, 2 ausência.

- Definições de códigos ou símbolos usados para registrar dados ausentes: número 2 refere-se a ausência

Banco tabela 3

- Número de variáveis: 11.

- Número de casos ou linhas: 402.

- Lista de variáveis, incluindo nomes completos e definições (soletrar palavras abreviadas) de cabeçalhos de coluna para dados tabulares:
- Aleitamento materno exclusivo (AME) aos 6 meses;
- Diarreia e gastroenterite de origem infecciosa presumível (Gastro 1);
- Doença de refluxo gastroesofágico (Gastro 2);
- Transtornos funcionais do intestino (Gastro 3);
- Infecções agudas das vias aéreas superiores de localizações múltiplas (Resp 1);
- Tosse (Resp 2);
- Otite média supurativa e as não especificadas (Resp 3);
- Dor abdominal e pélvica (Outros 1);
- Dermatite das fraldas (Outros 2);
- Febre de origem desconhecida (Outros 3);
- Outros transtornos do trato urinário (Outros 4).

- Unidades de medida
- Aleitamento materno exclusivo (AME) aos 6 meses: 1 presença, 2 ausência;
- Diarreia e gastroenterite de origem infecciosa presumível (Gastro 1): 1 presença, 2 ausência;
- Doença de refluxo gastroesofágico (Gastro 2): 1 presença, 2 ausência;
- Transtornos funcionais do intestino (Gastro 3): 1 presença, 2 ausência;
- Infecções agudas das vias aéreas superiores de localizações múltiplas (Resp 1): 1 presença, 2 ausência;
- Tosse (Resp 2): 1 presença, 2 ausência;
- Otite média supurativa e as não especificadas (Resp 3): 1 presença, 2 ausência;
- Dor abdominal e pélvica (Outros 1): 1 presença, 2 ausência;
- Dermatite das fraldas (Outros 2): 1 presença, 2 ausência;
- Febre de origem desconhecida (Outros 3): 1 presença, 2 ausência;
- Outros transtornos do trato urinário (Outros 4): 1 presença, 2 ausência.

- Definições de códigos ou símbolos usados para registrar dados ausentes: número 2 refere-se a ausência
